# Urinary 4-pyridoxic acid as a non-invasive biomarker for evaluating osteoarthritis severity: findings from the ROAD study

**DOI:** 10.1007/s40520-025-02944-6

**Published:** 2025-02-25

**Authors:** Noriko Yoshimura, Aiko Miyazaki, Toshiko Iidaka, Nobuo Ando, Gaku Tanegashima, Shigeyuki Muraki, Horiyuki Oka, Sakae Tanaka

**Affiliations:** 1 Department of Preventive Medicine for Locomotive Organ Disorders, 22ndMedical and Research Center, The University of Tokyo, 7-3-1 Hongo, Bunkyo-Ku, Tokyo 113-8655 Japan; 2 Fushimi Pharmaceutical Co., Ltd, Marugame, Kagawa Prefecture Japan; 3 Department of Orthopaedic Surgery, The University of Tokyo, Tokyo, Japan; 4 Division of Musculoskeletal AI System Development, Faculty of Medicine, The University of Tokyo, Tokyo, Japan

**Keywords:** 4-pyridoxic acid, Knee osteoarthritis, Lumbar spondylosis, Population-based cohort ROAD, Predictive biomarker

## Abstract

**Background:**

The early detection of osteoarthritis (OA) requires reliable biomarkers; however, reports identifying such biomarkers remain limited.

**Aims:**

This study aimed to evaluate the potential of urinary 4-pyridoxic acid (4PA) as a biomarker for the severity of knee osteoarthritis (KOA) and lumbar spondylosis (LS) in Japanese adults, using data from the population-based cohort study.

**Methods:**

Data were analysed from 1566 participants (510 men and 1,056 women) aged ≥ 40 years, who were enrolled in the Research on Osteoarthritis/Osteoporosis against Disability (ROAD) cohort, a population-based study initiated in 2005. Participants underwent radiographic assessments of the knees and lumbar spine, and urinary 4PA levels were measured using high-performance liquid chromatography. Logistic regression analyses were performed to evaluate the association between urinary 4PA levels and the Kellgren–Lawrence (KL) grade of KOA and LS, adjusting for age, sex, body mass index (BMI), and lifestyle factors.

**Results:**

Urinary 4PA levels were significantly higher in participants with KL grade 4 KOA compared to those with lower KL grades (p < 0.001). This association remained significant after adjusting for confounding factors. In contrast, no significant differences in 4PA levels were observed across the KL grades for LS, although a slight increase in 4PA levels was noted in KL grade 4 cases.

**Discussion and conclusions:**

These findings suggest that urinary 4PA could serve as a biomarker for assessing KOA severity, particularly in advanced stages. While the detection of early OA using 4PA remains challenging, the significant increase in KL grade 4 cases highlights its potential role in guiding treatment decisions, such as surgical intervention.

## Introduction

According to the 2023 abridged life tables from the Ministry of Health, Labour and Welfare, the average lifespan in Japan is 81.09 years for men and 87.14 years for women. Considering that when surveys began in 1947, the remaining life expectancy was 50.06 years for men and 53.96 years for women, the lifespan of Japanese people has extended by more than 30 years over the past 77 years [1]. This extension in lifespan has propelled Japan to become the country with the highest life expectancy in the world. At the same time, it has also highlighted the issue of elderly care needs. According to the Long-Term Care Insurance Business Situation Report (2024, provisional version) [2], the number of individuals certified as requiring care (including those needing support) has significantly increased from 2.56 million in 2000 to 6.94 million at the end of 2022.

Regarding the reasons for becoming disabled and requiring care or support, the Ministry of Health, Labour and Welfare reported in its most recent National Livelihood Survey [3] that joint disorders rank fifth among the causes. However, when focusing specifically on the reasons for requiring support, joint disorders consistently ranked first [3]. This suggests that osteoarthritis (OA) the most common joint disorder, is frequently observed in the early stages of care dependency. Therefore, early detection and preventive measures against these conditions can significantly improve the quality of life for older adults. However, early detection of OA requires biomarkers that can accurately predict the condition, and there have been very few reports on such biomarkers.

In our search for novel biomarkers related to OA, we focused on urinary 4-pyridoxic acid (4PA). Vitamin B6 exists in three forms (pyridoxal, pyridoxine, and pyridoxamine), each with its active form, and is eventually excreted in the urine as 4PA. The active form, pyridoxal-5ʹ-phosphate, is involved in over 100 catalytic reactions and regulates various physiological processes [4]. Previous studies have shown a correlation between vitamin B6 and inflammatory markers in the body [5–7], and its relationship with OA via homocysteine metabolism has also been investigated [8, 9]. However, to date, no studies have directly elucidated the relationship between urinary 4PA and OA. In the present study, we hypothesizes that urinary 4PA levels correlate with the severity of osteoarthritis, reflecting its potential as a non-invasive biomarker. This study was planned to investigate 4PA’s effectiveness given the increasing need for reliable biomarkers in osteoarthritis management and prognosis.

We initiated a population-based prospective cohort study entitled the Research on Osteoarthritis/Osteoporosis against Disability (ROAD) in 2005 [10, 11] and completed 17 years of follow-up by the end of 2023. Using the information of the participants in the ROAD study 3rd survey conducted between 2012–2013, we examined the value of 4PA as a urinary biomarker in association with the presence of radiographic knee osteoarthritis (KOA) or lumbar spondylosis (LS) in older Japanese individuals residing in a rural community.

## Methods

This study, conducted with a cross-sectional design, utilized data from the third survey (2012–2013) of the population-based longitudinal ROAD cohort, which was followed from 2005 to 2023, in order to elucidate the utility of 4-Pyridoxic Acid in OA.

### Participants of the ROAD Study

The ROAD study, initiated in 2005, is a nationwide longitudinal cohort study that includes diverse regions across Japan, such as Itabashi in Tokyo, Hidakagawa in Wakayama’s mountainous regions, and the coastal area of Taiji in Wakayama. The project has collected clinical and genetic data from 3040 residents aged between 23 and 95, documented in previous publications [10, 11]. All participants provided written informed consent, and the study was approved by the ethics committees of the University of Tokyo and Wakayama Medical University (University of Tokyo, No. 1264 and 1326, and Wakayama Medical University, No. 373).

The third survey, conducted in 2012–2013, targeted previous participants and new ones aged 40 or older, using promotional efforts by local authorities. The survey included 2566 residents (urban: 845, mountainous: 769, coastal: 952). Urine tests for 4-Pyridoxic Acid analysis were only performed in the mountainous and coastal areas, as no urine tests were conducted in the urban area. From the mountainous and coastal regions, data from 1566 participants who provided urine samples were analyzed. The demographic characteristics of these participants are summarized in Table 1.

**Table 1:** Baseline characteristics of participants

		Total	Men	Women	Men vs Women
Variables	Unit	N=1566	N=510	N=1056	p values
age	years	65.6 (13.0)	66.2 (13.7)	65.3 (12.6)	0.190
weight	kg	56.4 (11.3)	64.5 (11.4)	52.5 (8.8)	<0.0001***
height	cm	156.2 (9.2)	164.9 (7.3)	151.9 (6.7)	<0.0001***
BMI	kg/m^2^	23.0 (3.5)	23.6 (3.4)	22.7 (3.5)	<0.0001***
coastal area	%	54.5	51.8	55.9	0.126
current smoker	%	8.8	20.7	2.9	<0.001***
current drinker	%	40.2	67.4	27.0	<0.001***
Urine examination					
4PA μmol/mmolCre		3.16 (11.93)	2.65 (12.28)	3.40 (11.75)	0.242
Osteoarthritis (knee, lumbar spine)					
KL grade (worse side, knee)		1.4 (1.3)	1.1 (1.2)	1.6 (1.3)	<0.0001***
KOA (either knee KL>=2, %)		41.1	29.5	46.8	<0.001***
KL grade (worst region, lumbar spine		2.5 (1.1)	2.6 (1.0)	2.4 (1.1)	0.0061**
LS (either lumbar KL>=2, %)		74.2	85.4	68.8	<0.001***

*BMI* Body mass index, *4PA*, 4-Pyridoxic Acid

*KL grade* Kellgren-Lawrence grade, *KOA* Knee osteoarthritis, *LS* Lumbar spondylosis

*p<0.05, **p<0.01. ***p<0.001

### Radiographic assessment

All participants underwent radiographic examination of both knees using an anteroposterior (AP) view with weight bearing and foot map positioning. Fluoroscopic guidance with a horizontal AP X-ray beam was used to properly visualise the joint space, and images were downloaded as Digital Imaging and Communications in Medicine files.

The severity of radiographic OA was determined according to the Kellgren–Lawrence (KL) grading as follows [12]: KL0, normal; KL1, slight osteophytes; KL2, definite osteophytes; KL3, joint or intervertebral space narrowing with large osteophytes; and KL4, bone sclerosis, joint or intervertebral space narrowing, and large osteophytes.

In the ROAD study, participants were classified as KL3 if they had joint or intervertebral space narrowing without the presence of large osteophytes. Radiographs of each site (knees, hips, and vertebrae) were examined by a single experienced orthopaedic surgeon, S.M., who was blinded to the participants’ clinical statuses. This approach ensured consistency and reduced variability in assessing the severity of OA. He was blinded to participants’ clinical statuses. If at least one knee joint was graded as KL2 or higher, the participant was diagnosed with radiographic KOA. Individuals who underwent knee arthroplasty were excluded from the analysis. Similarly, if at least one intervertebral level of the lumbar spine was graded as KL2 or higher, the participant was diagnosed with radiographic LS.

### Measurements of 4PA

The 4PA concentration in the subjects’ urine samples was measured using the following four steps:

#### Chemicals and reagents

The 4PA (P9630) was obtained from Sigma-Aldrich (St. Louis, MO, USA). Ethylenediaminetetraacetic acid disodium salt (EDTA-2Na) dihydrate (000–29135), sodium dihydrogen phosphate (000–72525), disodium hydrogen phosphate (000–72555), and acetonitrile (000–00403) were obtained from KISHIDA CHEMICAL Co. LTD (Osaka, Japan). Methanol (106,007) was obtained from Merck (Darmstadt, Germany). Activated charcoal (C2194) was obtained from Tokyo Chemical Industry Co., Ltd. (Tokyo, Japan) to prepare blank samples. Ultrapure water was prepared using the Synergy UV system (Millipore, Bedford, MA, USA).

#### Standard samples, calibration curves, and quality control samples

The 4PA measurement was adapted from the methods of Tawar et al. [13] and Akiyama et al. [14]. The standard stock solution of 4PA was dissolved in ultrapure water to a final concentration of 250 μmol/L and stored frozen until analysis.

Standard solutions of 12 concentrations (0, 1, 2.5, 5, 10, 25, 50, 100, 200, 300, 400, and 500 nmol/L) were prepared by diluting the stock solutions with 60 mmol/L phosphate buffer (pH 7.0). Calibration curves were obtained by plotting the peak areas of 4PA against the corresponding concentrations. Quality control (QC) samples (urine) were prepared from blank urine samples. Blank urine was prepared as follows. Activated charcoal was washed with acetonitrile, rinsed thoroughly with ultrapure water, and dried. Washed activated charcoal (10 g) was added to 100 mL of urine and reacted with shaking overnight. A portion of the reaction solution was tested for the presence of the 4PA peak using high-performance liquid chromatography (HPLC). If no 4PA peak was detected, the reaction was terminated; if a peak was detected, additional washed activated charcoal was added, or the reaction time was extended. QC samples (urine) were prepared by diluting 250 μmol/L 4PA standard stock solution with 60 mmol/L phosphate buffer to make 50 nmol/L, 100 nmol/L, 1 μmol/L, 2.5 μmol/L, and 4 μmol/L QC stock solutions. Each QC stock solution was mixed at a ratio of 1:1:8 (QC stock solution: blank urine: 60 mmol/L phosphate buffer [pH 7.0]) to prepare QC solutions (final concentrations: 5, 10, 100, 250, and 400 nmol/L).

#### Urine samples

Urine samples were diluted tenfold with 60 mmol/L phosphate buffer (pH 7.0), filtered through a 0.2 µm filter, and measured using HPLC. As a general protocol, 3 μL of urine was mixed with 27 μL of 60 mmol/L phosphate buffer (pH 7.0), filtered through a 0.2 μm filter, and measured using HPLC. Samples with values exceeding 500 nmol/L were re-diluted to below 500 nmol/L and re-measured. The measured values of the urine samples were corrected using urinary creatinine, and urinary 4-PA levels were obtained.

#### HPLC system and chromatographic Conditions

The HPLC system used was the Prominence LC-20A series (CBM-20A, LC-20AD, SIL-20AC, CTO-20AC, RF-20A xs, DGU-20A5R; Shimadzu, Japan). In the assessment, 4PA was detected at Ex 320 nm and Em 422 nm, and the column oven was set at 40℃. The autosampler temperature was set at 4℃. Separation was performed using a YMC-Triart C18 column (3 μm, 100 × 4.6 mm, YMC, Japan).

Mobile phase A was methanol, mobile phase B was 60 mM phosphate buffer (pH 7.0) and 0.05% EDTA-2Na, and mobile phase C was 60% acetonitrile and 0.1% EDTA-2Na. The injection volume was 20 μL. Samples were analysed using HPLC. The total analysis time was 56 min, and the flow rate was set between 0.8 and 2.0 mL/min. The flow rate was maintained at 0.8 mL/min for the first 3.2 min, increased to 1.2 mL/min (3.2–19 min), then to 1.5 mL/min (19–28 min), and finally to 2.0 mL/min (28–43 min). The flow rate was reduced to 1.8 mL/min (43–46 min) and equilibrated at 0.8 mL/min (46–56 min).

The mobile phases consisted of A (1.5–100%), B (0–98.5%), and C (0–100%), and the gradient conditions were as follows: for the first 11 min, A was maintained at 1.5% and B at 98.5%. From 11 to 18 min, A increased linearly to 20%, whereas B decreased to 80%. From 18 to 24 min, A was maintained at 20% and B at 80%; by 31 min, B was reduced to 0%, while A was increased to 100%; from 36 to 41 min, C was maintained at 100%; and finally, from 41 to 56 min, A and B returned to 1.5% and 98.5%, respectively, for equilibration. Washing was performed at each stage, and the column conditions were set in the following order: analysis, washing, and equilibration.

Analyses were performed in the following order: standard solution, QC samples, urine samples, and QC samples. If the QC sample was within the control range, the measured data were considered acceptable.

### Statistical analyses

All statistical analyses were performed using the STATA software (STATA, College Station, Texas, USA). Differences in proportions were compared using the chi-squared test. The significance of the differences in continuous variables was evaluated using analysis of variance for comparisons among multiple groups or Scheffe’s least significant difference test for pairwise group comparisons. All p-values and 95% confidence intervals were two-sided. Statistical significance was set at p < 0.050.

Logistic regression analysis was used to test the association between 4PA and the presence of KOA and LS. In the analysis, the KL grade of KOA or LS (0: absence, 1: presence) was used as the dependent variable, and 4PA values were used as independent variables after adjusting for age (+ 1 year), sex (0: men; 1: women), Body mass index (BMI; + 1 kg/m^2^), regional differences (0: mountainous area; 1: coastal area), and smoking and drinking status.

## Results

### Background characteristics of study participants

As noted in the Methods section, 2566 residents from urban, mountainous, and coastal areas participated in the third visit of the ROAD study. Among the health examinations conducted in these three regions, urine tests were performed only in mountainous and coastal areas. Therefore, participants from urban areas were excluded. Of the 1,721 participants in the third survey conducted in mountainous and coastal areas, data from 1566 participants (510 men and 1,056 women) who provided urinary samples for the 4PA measurements were used. The remaining participants did not provide their urine samples.

Table 1 presents the selected characteristics of the participants classified by sex, including age, height, weight, BMI, and KL grades of the knee and lumbar spine. Significant gender differences were observed in the proportion of KL ≥ 2 grades for the knee and lumbar spine; KOA was significantly more prevalent in women, while LS was more common in men (p < 0.001).

### Relationship between 4PA values and KL grade of the knee

**Fig. 1:**
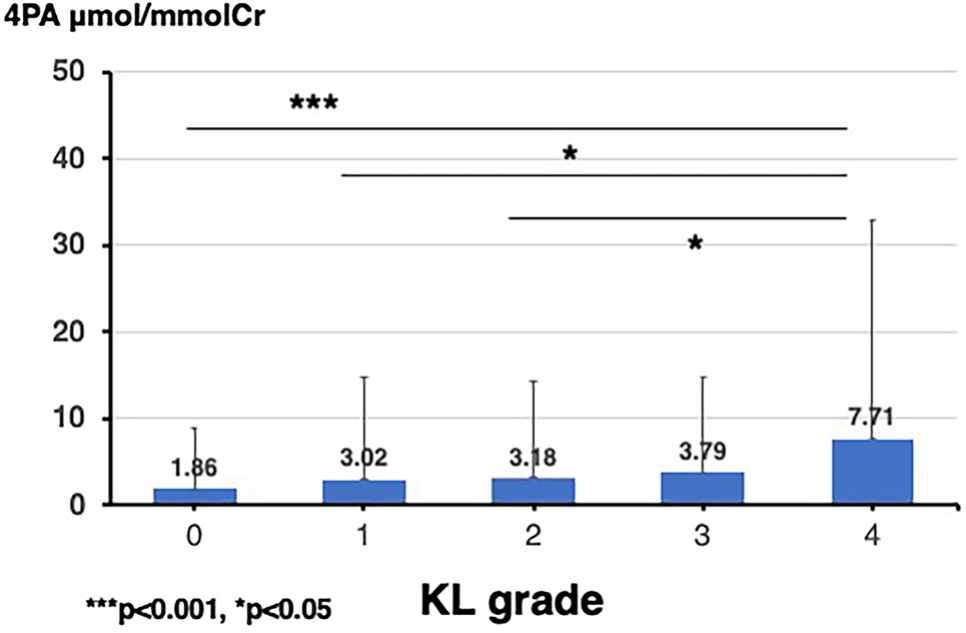
Mean levels of 4-pyridoxic acid by Kellgren-Lawrence grade in patients with knee osteoarthritis

**Fig. 2:**
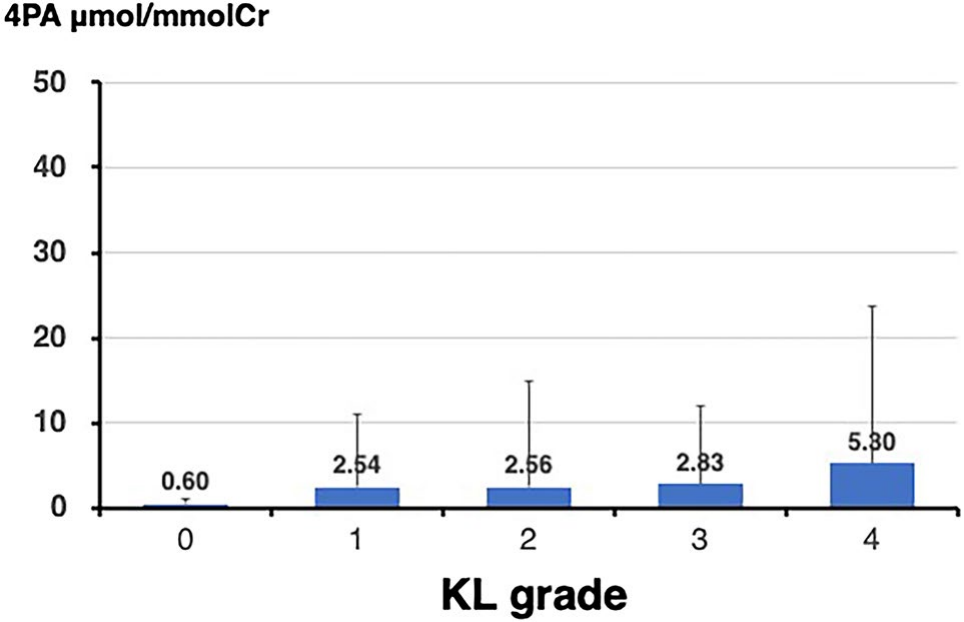
Mean levels of 4-pyridoxic acid by Kellgren-Lawrence grade in patients with lumbar spondylosis

Figures 1 and 2 show the mean 4PA values classified by the KL grade for the knee and lumbar spine, respectively. Regarding the association between OA severity and 4PA, 4PA values were found to increase significantly with higher KL grades for both KOA and LS (KOA, p < 0.001; LS, p < 0.050). A comparison of the 4PA values between each pair of KL grades for the knee showed that the 4PA values for KL grade 4 were significantly higher than those for the other KL grades (KL 4 vs KL 0, p < 0.001; KL 4 vs KL 1, 2, and 3, each p < 0.050). Contrastingly, a comparison of the 4PA values between each pair of KL grades for the lumbar spine showed no significant differences, although a weak trend was observed, with KL grade 4 tending to be higher than KL grades 1, 2, and 3 (KL grade 4 vs KL grade 1, p = 0.065; KL grade 4 vs KL grade 2, p = 0.090; KL grade 4 vs KL grade 3, p = 0.091).

Tables 2 and 3 present the urinary concentrations of 4PA classified by the KL grade, along with baseline characteristics such as sex, age, height, weight, BMI, and the prevalence of current smoking and drinking. As the KL grade of the knee increased, the mean age, BMI, proportion of women, and proportion of participants from mountainous areas also increased. The proportion of current smokers and drinkers was the lowest at KL grade 4. Similarly, as the KL grade of the lumbar spine increased, the mean age and BMI tended to increase, and the proportion of drinkers was lowest at KL grade 4. While other variables showed differences across groups, a consistent trend based on an increase in KL grades was not observed.

**Table 2:** Comparison of urinary concentration of 4PA and selected characteristics classified by KL grades of the knee

	KL 0 N=477	KL 1 N=438	KL 2 N=266	KL 3 N=268	KL 4 N=105	Group differences p values
Urine examination						
4PA (μmol/mmolCre)	1.86 (7.02)	3.02 (11.85)	3.18 (11.15)	3.78 (11.15)	7.70 (25,30)	0.0002***
age (years)	55.5 (12.7)	65.8 (10.5)	70.7 (9.4)	73.9 (8.4)	76. 7 (7.7)	<0.0001***
height (cm)	160.9 (8.7)	156.8 (8.4)	153.2 (8.2)	152.1 (8.5)	149.8 (8.0)	<0.0001***
weight (kg)	58.6 (11.7)	55.6 (11.3)	54.5 (10.1)	56.1 (11.3)	55.5 (10.7)	<0.0001***
BMI (kg/m^2^)	22.5 (3.3)	22.5 (3.5)	23.1 (3.1)	24.2 (4.0)	24.7 (3.8)	<0.0001***
% female	56.8	65.3	74.8	77.2	79.1	<0.001***
% seaside town	67.9	59.4	41.0	39.9	45.7	<0.001***
% current smoker	15.8	7.3	4.1	5.6	2.9	<0.001***
% current drinker	53.5	39.5	37.2	30.2	16.2	<0.001***

*4PA*, 4-Pyridoxic Acid; *BMI* Body mass index

*KL grade* Kellgren-Lawrence grade

*p<0.05, **p<0.01. ***p<0.001

**Table 3:** Comparison of urinary concentration of 4PA and selected characteristics iclassified by KL grades of the lumbar spine

	KL 0 N=11	KL 1 N=389	KL 2 N=327	KL 3 N=538	KL 4 N=286	Group differences p values
Urine examination						
4PA (μmol/mmolCre)	0.60 (0.41)	2.54 (8.46)	2.57 (12.44)	2.83 (9.13)	5.30 (18.34)	0.0174*
age (years)	50.5 (20.0)	56.1 (13.2)	66.2 (11.9)	68.9 (10.5)	72.1 (9.8)	<0.0001***
height (cm)	155.7 (5.1)	156.9 (8.5)	159.2 (9.8)	154.5 (8.5)	154.7 (9.8)	<0.0001***
weight (kg)	50.4 (6.9)	55.1 (11.1)	59.5 (12.4)	55.6 (10.5)	56.5 (11.0)	<0.0001***
BMI (kg/m^2^)	20.8 (3.1)	22.3 (3.4)	23.3 (3.6)	23.2 (3.6)	23.5 (3.4)	<0.0001***
% female	90.9	81.2	45.3	72.1	63.6	<0.001***
% seaside town	54.6	61.4	53.5	50.0	54.9	0.017*
% current smoker	18.2	6.4	15.6	6.0	9.1	<0.001***
% current drinker	63.6	39.3	49.5	36.9	35.3	0.001***

*4PA* 4-Pyridoxic Acid, *BMI* Body mass index

*KL grade* Kellgren-Lawrence grade

*p<0.05, **p<0.01. ***p<0.001

### Further analysis of 4PA values and KL grades

Figures 1 and 2 show the mean 4PA values classified by the KL grade for the knee and lumbar spine, respectively. As mentioned earlier, the 4PA values increased significantly with higher KL grades for both KOA and LS (KOA, p < 0.001; LS, p < 0.050). A comparison of the 4PA values between each pair of KL grades for the knee showed that the 4PA values for KL grade 4 were significantly higher than those for the other KL grades (KL 4 vs KL 0, p < 0.001; KL 4 vs KL 1, 2, and 3, each p < 0.050). For the lumbar spine, no significant differences were found between each pair of KL grades, although a slight upward trend was observed between KL grade 4 and KL grades 1, 2, and 3 (KL 4 vs KL 1, p = 0.065; KL 4 vs KL 2, p = 0.090; and KL 4 vs KL 3, p = 0.091).

### Multinomial logistic regression analysis

To identify the significance of urinary 4PA concentrations in relation to OA severity, a multinomial logistic regression analysis was performed. In the analysis, the KL grades of KOA or LS were used as the dependent variables (0: KL grades 1, 2, 3; 1: KL 0; 2: KL 4), and 4PA values were used as the independent variables, adjusting for age (+ 1 year), sex (0: men; 1: women), BMI (+ 1 kg/m^2^), regional differences (0: mountainous area; 1: coastal area), smoking status (0: non-smoker; 1: current smoker), and drinking status (0: non-drinker; 1: current drinker). Table 4 shows the association between KL severity and urinary 4PA concentration. Compared to the groups with KL grades 1, 2, and 3 of the knee, urinary 4PA values were significantly higher in the KL grade 4 group after adjusting for confounding factors, whereas no significant association was found with KL grade 0. This trend was also observed in the KL grades of the lumbar spine.

**Table 4:** Association between KL severity and urinary concentration of 4PA

Knee vs KL 1,2,3	KL0		vs KL 1,2,3	KL 4		vs KL 1,2,3
	RRR	95% CI	p values	RRR	95% CI	p values
Explanatory variable						
urine 4PA (μmol/mmolCre)	1.00	0.94–1.01	0.807	1.01	1.00–1.03	0.016*
Adjusted variables						
age (+1 yr)	0.90	0.88–0.91	<0.001***	1.11	1.08–1.14	<0.001***
gender (0: men, 1:women)	0.32	0.23–0.45	<0.001***	1.71	0.97–2.99	0.063
BMI (+1kg/m2)	0.89	0.86–0.93	<0.001***	1.20	1.13–1.28	<0.001***
regional difference (0: mountainous, 1: coastal)	1.86	1.40–2.46	<0.001***	0.85	0.55–1.32	0.480
current smoking (0: no, 1: yes)	0.98	0.62–1.55	0.934	1.52	0.43–5.41	0.518
current drinking (0: no, 1: yes)	1.13	0.84–1.53	0.416	0.43	0.24–0.79	0.006**

Lumbar spine vs KL 1,2,3	KL0		vs KL 1,2,3	KL 4		vs KL 1,2,3
	RRR	95% CI	p values	RRR	95% CI	p values
Explanatory variable						
urine 4PA (μmol/mmolCre)	0.59	0.17–2.01	0.399	1.01	1.00–1.02	0.036*
Adjusted variables						
age (+1 yr)	0.95	0.90–0.99	0.030*	1.07	1.05–1.08	<0.001***
gender (0: men, 1:women)	7.42	0.78–70.6	0.081	0.96	0.70–1.32	0.802
BMI (+1kg/m^2^)	0.84	0.67–1.04	0.110	1.07	1.02–1.10	0.001**
regional difference (0: mountainous, 1: coastal)	0.65	0.18–2.36	0.515	1.32	1.00–1.74	0.050
current smoking (0: no, 1: yes)	2.73	0.48–15.6	0.259	1.94	1.16–3.23	0.011*
current drinking (0: no, 1: yes)	2.44	0.03–7509.4	0.676	0.98	0.71–1.35	0.897

*4PA* 4-Pyridoxic Acid; *BMI* Body mass index

*RRR* Ralative risk ratio, *95% CI* 95% confidence interval

*p<0.05, **p<0.01. ***p<0.001

## Discussion

In the present study, we observed that urinary 4PA levels were significantly higher in participants with advanced KOA (KL grade 4) than in those with lower grade KOA. This association was robust and persisted even after adjusting for potential confounders such as age, sex, BMI, and lifestyle factors. These findings suggest that 4PA could serve as a valuable biomarker for assessing the severity of OA, particularly in advanced stages.

As the final metabolite of vitamin B6, 4PA is excreted from the body via the kidneys in urine. In the body, the active form of vitamin B6 is involved in more than 100 reactions of great variety, and its role is significant [4]. The relationship between vitamin B6 and inflammation has been reported in several studies; for example, Morris et al. reported the relationship between vitamin B6 intake and serum C-reactive protein (CRP) levels, a marker of inflammation, in 2,686 participants of the 2003–2004 National Health and Nutrition Examination Survey in the USA [6]. Analysis showed a relationship between higher total vitamin B6 intake and lower serum CRP concentrations, and results from other studies suggest that inflammation may impair vitamin B6 metabolism. Similarly, the Framingham Offspring Study confirmed an association between vitamin B6 status and total inflammation scores based on 13 inflammation markers and reported an inverse correlation between active vitamin B6 and inflammation scores [6]. Furthermore, the WENBIT study showed that systemic inflammation promotes the degradation of active vitamin B6 to 4PA [15]. These reports suggest that vitamin B6 plays an important role in inflammation and is an important factor in OA.

Exercise has also been suggested to improve joint function and reduce pain [16], and guidelines on the type and frequency of exercise have been reported [17]. To investigate the effects of exercise on chondrocytes in this context, Carbonare et al. found that osteogenic genes were upregulated in progenitor cells treated with serum collected before and after exercise, improving bone differentiation and regulating various aspects such as immune responses [18]. Interestingly, in the second report of the study, Deiana et al. similarly analysed human serum before and after exercise and found that the expression of genes related to chondrogenic differentiation was increased after exercise and that metabolites related to vitamin B6 were highly regulated. Further in vitro studies to assess the effects of vitamin B6 on chondrocytes from these results also showed that vitamin B6 increased chondrogenesis genes, counteracted the effects of the inflammatory cytokine IL-1β, suppressed oxidative stress, and reduced apoptosis. The results of this study were consistent with those of the National Institutes of Health. To our knowledge, this is the first report showing that exercise protects cartilage by activating vitamin B6 metabolism [19]. This result is noteworthy, as the majority of vitamin B6 in the body is bound to glycogen phosphorylase and stored in muscle [20]. In a more recent report, Fangra corroborated these reports using a mouse model of OA and investigated the administration of vitamin B6 to the model mice, which improved OA severity, maintained cartilage thickness, suppressed inflammatory cytokine expression and apoptosis, and improved extracellular matrix metabolism. These vitamin B6 results are common in both in vivo and in vitro experiments and are consistent with previous reports [21]. In addition to these reports, urinary metabolic analysis of rat OA models has also confirmed an increase in 4PA compared to that in controls [22] and other reports have shown that vitamin B6 metabolism is regulated during injury in mouse articular cartilage [23].

Our findings underscore the potential clinical applications of measuring urinary 4PA levels. As a non-invasive biomarker, 4PA can help stratify patients according to the severity of their condition, facilitating tailored therapeutic interventions. Moreover, monitoring changes in the 4PA levels over time could serve as a tool for evaluating the effectiveness of treatment strategies, thus optimising patient management.

However, the cross-sectional nature of this study limits our ability to draw causal inferences between the 4PA levels and OA progression. Longitudinal studies are necessary to confirm whether elevated 4PA levels can predict the progression of OA. Importantly, our ROAD study conducts longitudinal follow-ups, which will enable us to explore the impact on the incidence and worsening of the condition in a longitudinal framework. Additionally, although we adjusted for several known confounders, other unmeasured variables may have influenced the observed relationships. The geographical and demographic limitations of the study population restrict the generalisability of our findings to other populations and regions. Future research should include a more diverse cohort and consider a wider range of metabolic and inflammatory markers to validate and enhance the clinical utility of 4PA.

Future research should focus on validating these findings in larger and more diverse populations and exploring the mechanisms linking vitamin B6 metabolism with OA progression. Upon validation, urinary 4PA could be developed as a useful biomarker for assessing and monitoring the severity of OA in clinical practice.

## Conclusions

Our study highlights the potential of urinary 4PA levels as biomarkers for OA severity, particularly in the knee and lumbar spine. These findings pave the way for further research into the predictive power of 4PA in OA, with the ultimate goal of improving the diagnostic accuracy and therapeutic outcomes for patients with this debilitating condition.

## Data Availability

No datasets were generated or analysed during the current study.
